# Filamentous white matter prion protein deposition is a distinctive feature of multiple inherited prion diseases

**DOI:** 10.1186/2051-5960-1-8

**Published:** 2013-05-09

**Authors:** Lilla Reiniger, Ilaria Mirabile, Ana Lukic, Jonathan DF Wadsworth, Jacqueline M Linehan, Michael Groves, Jessica Lowe, Ronald Druyeh, Peter Rudge, John Collinge, Simon Mead, Sebastian Brandner

**Affiliations:** Division of Neuropathology, National Hospital for Neurology and Neurosurgery, Queen Square, London UK; Department of Neurodegenerative Disease, UCL Institute of Neurology, Queen Square, London UK; National Prion Clinic, National Hospital for Neurology and Neurosurgery, Queen Square, London UK; MRC Prion Unit, UCL Institute of Neurology, London, UK

**Keywords:** Creutzfeldt-Jakob disease, Inherited prion disease, White matter, *PRNP* gene mutation, Octapeptide repeat insert mutation, OPRI, Gerstmann Sträussler Scheinker Syndrome, GSS, Transgenic mice, Axons, Myelin basic protein

## Abstract

**Background:**

Sporadic, inherited and acquired prion diseases show distinct histological patterns of abnormal prion protein (PrP) deposits. Many of the inherited prion diseases show striking histological patterns, which often associate with specific mutations. Most reports have focused on the pattern of PrP deposition in the cortical or cerebellar grey matter.

**Results:**

We observed that the subcortical white matter in inherited prion diseases frequently contained filamentous depositions of abnormal PrP, and we have analysed by immunohistochemistry, immunofluorescence and electron microscopy 35 cases of inherited prion disease seen at the UK National Prion Clinic. We report here that filamentous PrP is abundantly deposited in myelinated fibres in inherited prion diseases, in particular in those with N-terminal mutations.

**Conclusions:**

It is possible that the presence of filamentous PrP is related to the pathogenesis of inherited forms, which is different from those sporadic and acquired forms.

## Background

Human prion diseases represent a clinically and pathologically diverse group of neurodegenerative disorders which invariably progress to a fatal outcome. Prion diseases comprise sporadic forms (Creutzfeldt-Jakob disease (sCJD)), inherited prion diseases (IPD) (sometimes also classified as familial CJD (fCJD), Gerstmann-Sträussler-Scheinker Syndrome (GSS) and fatal familial insomnia (FFI)), and acquired forms with variant CJD (vCJD), iatrogenic CJD and kuru. The common neuropathological characteristics of human prion diseases are intraneuronal vacuolation with neuronal loss (spongiform degeneration) in the grey matter, reactive astrocytosis and microglia activation as well as a highly variable accumulation of abnormal prion protein (PrP^Sc^), which represents misfolded forms of normal cellular prion protein (PrP^C^). The patterns of PrP^Sc^ deposition in prion diseases are well characterised at immunohistochemical and ultrastructural levels [1, 2] and most observations focus on pathological changes in the grey matter. Involvement of the cerebral and cerebellar white matter in sCJD has been described and suggested to be a primary or secondary process in panencephalopathic type CJD [3, 4]. Cerebellar white matter PrP deposition is a prominent feature in the “VV2” subtype type [5]. It also occasionally occurs in other forms of prion diseases, where it is thought to be secondary to cortical degeneration and neuronal loss. The presence of PrP plaques in the white matter has been described in various plaque-forming types of CJD including variant CJD [6] and cases of sCJD with methionine homozygosity at codon 129 of the *PRNP* gene (*PRNP* 129MM) [7]. In a series of 20 autopsy cases of CJD, white matter deposition of abnormal PrP was examined in four cases by immunohistochemistry and transmission electron microscopy [8]. Abnormal PrP accumulation in the white matter is also seen in sheep with atypical scrapie [9] as well as in scrapie-infected Syrian hamsters where an intense white matter PrP^Sc^ signal was seen in histoblot studies. This was thought to support the hypothesis that PrP^Sc^ is transported along axons [10].

Having observed conspicuous filamentous deposits of abnormal PrP in the white matter in autopsy material from patients with inherited prion disease attending the National Prion Clinic and examined at autopsy between 2004 and 2012, we have subsequently analysed our cases systematically for the presence of white matter PrP deposits. We distinguished the patterns of small granular deposits or small plaques that are known for sCJD from a fine, thread like pattern that was seen in inherited cases. The present study describes the frequency, intensity and incidence of this pattern in a series of 35 cases of inherited prion diseases, comprising octapeptide repeat insertions (4OPRI, 5OPRI and 6OPRI) and *PRNP* point mutations of the codons 102, 117, 178, 200 and 210. In addition we give a brief overview of the salient PrP pathology that has been described previously in these inherited forms [11]. We have deliberately omitted a detailed description and discussion of other features such spongiform degeneration, neuronal loss, gliosis, and the glycotype patterns, as these have been extensively reported elsewhere.

## Methods

### Research governance, post mortem examination and tissue preparation

Ethical approval for these studies was obtained from the Local Research Ethics Committee of UCL Institute of Neurology/National Hospital for Neurology and Neurosurgery. Informed consent to use the tissue for research was obtained. Clinical data, disease history, *PRNP* mutation and *PRNP* codon 129 status were available for all cases. Autopsies were carried out in a post mortem room designated for high risk autopsies. Brains were removed and stored in buffered formalin. The frontal, temporal, parietal and occipital cortex and the cerebellum were dissected during the post mortem procedure and immersed in 10% buffered formalin for up to one week, immersed into 98% formic acid for one hour and postfixed for 24 h in 10% buffered formalin. Tissues were then processed through graded alcohols and embedded in paraffin wax. In a small subset of cases, a small tissue block was prepared from the frontal cortex and white matter and immersed into glutaraldehyde for preparation of semithin resin section and electron microscopy. Frozen tissue of frontal brain and cerebellum was collected routinely and banked. The remaining brains were stored permanently in 10% formalin and additional brain regions were dissected and processed to paraffin blocks at later time points.

### Antibodies and immunohistochemistry

In this study we used anti-PrP ICSM35 (D-Gen Ltd, London, UK, raised in *Prnp*^o/o^ mice against recombinant human PrP as previously described [12];) and anti PrP KG9 TSE Resource Centre, Roslin Institute University of Edinburgh [13]. ICSM35 recognises residues 93–102 and KG9 binds to residues 140–180 of human PrP. To detect abnormal PrP deposition, mounted sections were treated with 98% formic acid for 5 min, placed on an automated Ventana Discovery staining machine, heated to 95°C in 2.1 mM Tris–HCl, 1.3 mM EDTA, 1.1 mM sodium citrate, pH 7.8, for 30 min, digested for 16 min with a low concentration of protease (Protease 3, 0.02 U/ml alkaline protease, order no 760–2020 Ventana Medical Systems), incubated in Superblock for 10 min, then exposed to the primary antibody followed by biotinylated anti mouse IgG secondary antibody and visualised using the iView detection kit Haematoxylin was used as counterstain.

### Immunofluorescence

Immunofluorescent dual labelling was carried out on selected, representative frontal lobe sections of cases with 4OPRI, 6OPRI, P102L, A117V, D178N, E200K mutations and sCJD 129MM, MV, and VV. For dual labelling and co-localisation of abnormal PrP in white matter structures we applied the same antigen retrieval conditions as above. Then we dispensed the antibody ICSM35 (1:1500) together with either anti-Neurofilament (NF200, SIGMA N5389; 1:100) or anti-Myelin Basic Protein (SMI94, Covance, 1:250) followed by the secondary antibodies labelled with fluorochromes Alexa 488 (goat anti Mouse IgG1) or Alexa 546 (goat anti mouse IgG2b). Images were taken on a ZEISS LSM510Meta Laser Scanning confocal microscope, using a Plan-Apochromat 63×/1.4 Oil objective. All images were taken at a scanning resolution that matched the optical resolution of the lens (pixel size corresponding to 99.6 nm on the section).

### Transmission electron microscopy

Tissue blocks of no more than 2 mm^3^ were immersed in 2.5% glutaraldehyde, treated with formic acid for 24 h and following postfixation on glutaraldehyde, processed for resin (Epon) embedding. Resin sections were stained with Toluidine blue and suitable regions were selected for electron microscopy. Ultra-thin sections were stained with lead citrate and examined in a Jeol 100-CXII electron microscope. Images were recorded on a 4Megapixel SIS Megaview digital camera.

### Western blotting and glycotyping

Brain tissue homogenates (10% w/v) from frozen samples of frontal cortex were prepared in Dulbecco’s phosphate buffered saline lacking Ca^2+^ or Mg^2+^ ions using tissue grinders or by serial passage through needles of decreasing diameter. Aliquots (typically 20 μl) were removed and proteinase K added from a 1 mg/ml stock solution (prepared in water) to give a final concentration in the sample of 50 μg/ml. Following incubation at 37°C for 60 min, samples were centrifuged at 16100 g for 1 min before termination of the digestion by the addition of an equal volume of 2 × SDS sample buffer (125 mM Tris–HCl, pH 6.8, 20% v/v glycerol, 4% w/v sodium dodecyl sulphate, 4% v/v 2-mercaptoethanol, 0.02% w/v bromophenol blue) containing 8 mM 4-(2-aminoethyl)-benzene sulfonyl fluoride and immediate transfer to a 100°C heating block for 10 min. Samples were centrifuged at 16100 g for 1 min prior to electrophoresis in 16% tris-glycine gels (Invitrogen). Gels were electroblotted onto PVDF membrane (Immobilon-P; Millipore) and subsequently blocked in PBS containing 0.05% v/v Tween-20 (PBST) and 5% non-fat milk powder for 60 min. After washing in PBST, the membranes were incubated with anti-PrP monoclonal antibody 3F4 (0.2 μg/ml in PBST) before washing in PBST (60 min) and incubation with alkaline-phosphatase-conjugated goat anti-mouse IgG secondary antibody (Sigma-Aldrich Prod. No. A2179) diluted 1:10, 000 in PBST. After washing (1 h with PBST and 2 × 5 min with 20 mM Tris pH 9.8 containing 1 mM MgCl_2_) blots were incubated for 5 min in chemiluminescent substrate (CDP-Star; Tropix Inc) and visualized on Biomax MR film (Kodak). Control samples (of known PrP^Sc^ type) were analysed in parallel and run on the same blots to enable assignment of molecular strain type according to the London classification [14, 15].

### Examination and quantification

We analysed 35 autopsy cases of inherited prion disease seen at the National Prion Clinic, NHNN and these were compared with 26 cases with sCJD (9 cases with codon 129 MM, 10 with MV and 7 with VV polymorphism). The inherited cases included the following *PRNP* gene mutations (numbers of cases in brackets): 4 octapeptide/96 base pair repeat insertion [4OPRI] (3), 5OPRI/120 bp (1), 6OPRI/144 bp (4), P102L (10), A117V (4), D178N (3), E200K (9) and V210I (1).

Brains were examined in the following areas: Frontal, temporal, parietal, occipital and cerebellar cortex. More regions were available for some of the brains, sometimes with very extensive sampling, but we restricted our analysis to the above-mentioned representative areas of subcortical white matter. Using a semi-quantitative scoring scale (Table 1), all features were recorded for the cortical areas and the cerebellum. Intensity and the deposition pattern of PrP were recorded for the cortical grey matter and the subcortical white matter, as well as the cerebellar molecular and granular layers and the cerebellar white matter. The following features were observed and recorded: in the cortical grey matter, there was synaptic labelling and deposition of plaques, in addition filamentous PrP was present in the white matter fibres extending into the cortex. In the white matter, we recorded small granules as well as thin threads of variable length, which depended on the orientation of the white matter tracts. The scores 0-3 reflect the abundance of PrP deposits (threads or granules), which is listed in Table 1. The quantity and the pattern of cortical prion pathology was recorded for all cases as shown in Table 2.

**Table 1:** **Description and definition of the quantification of the filamentous or granular depositions**

Score (granules or threads)	Number of inclusions (High power field (HPF) with a 40× objective)
0	Less than 1 inclusion per 4 HPF
1	More than 1 inclusion per 4 HPF, up to 5 inclusions per 1 HPF
2	5-20 inclusions per 1 HPF
3	More than 20 inclusions per 1 HPF

Scoring scheme used to semiquantitatively assess the frequency and density of granular or filamentous inclusions in DAB stained paraffin sections on a LEICA DM2500 with a 40× HCX Pan Apochromat objective.

**Table 2:** **List of all cases analysed for white matter PrP in this study**

Figure	Case no	Mutation	Codon 129 status	PrP^Sc^molecular strain type*	Gender	Age at death	Disease duration (mo)	Amount of PrP^Sc^in WM		PrP pattern WM T = Threads D = Dots		Other pathology	
								**FB**	**Cer**	**FB**	**Cer**	**Cortical PrP pattern**	**Spongiosis**
	1	V210L	MV	2	M	69	5	+	—	T, D	—	S1, PN1, D2	+
	2	E200K	MM	2	M	65	4	+	—	D	—	S2, GR1, PN1	+
	3	E200K	MV	ND	M	67	24	+	—	D	—	S1	+
	4	E200K	MM	ND	F	57	6	++	+	D	D	S1, PN1	+
Figure 2D-F, Figure 4A, B	5	E200K	MV	2/3	F	45	38	+++	+	T, D	D	S3, GR2	+++
	6	E200K	MM	ND	M	61	6	++	+	D	D	S2, GR2, PV2	+
	7	E200K	MM	2	M	65	5	—	—	—	—	S1, PN1	++
	8	E200K	MM	2	M	65	9	+	—	D		S1	+
	9	E200K	MM	2	F	69	4	+	+	D	—	S2, PN1	++
	10	E200K	MM	ND	M	47	19	++	++	T, D	T, D	S2, D1, PV2	+
	11	D178N	MM	Negative	M	60	7	+++	+	D	D	P3, D2	o
Figure 2A-C, Figure 3I, J	12	D178N	MV	ND	M	60	24	++	—	D	—	S2, GR1, P1	+++
	13	D178N	MV	ND	F	51	20	—	—	—	—	S1, GR1	++
	14	A117V	MV	Negative	M	43	6	+++	++	T	D	S2, P3, D3	o
	15	A117V	VV	Negative	M	40	46	+++	++	T	D	S2, P3	++
Figure 1M-P, Figure 3G, H	16	A117V	MV	Negative	M	47	47	+++	—	T	—	S1, P3	o
Figure 5C, D	17	A117V	MV	ND	F	40	98	+++	+	T	T, D	S2, P3	++
	18	P102L	MV	Negative	M	53	64	++	++	T	T, D	P3, D2	o
	19	P102L	MM	1	M	58	53	+	+	T	D	P3, D2	++
	20	P102L	MM	ND	M	64	24	—	+	—	T, D	S1, P1	o
Figure 1I-L	21	P102L	MM	ND	M	59	49	+++	+++	T, D	T, D	P3, D2	++
	22	P102L	MV	2	F	64	33	+++	++	D	D	S2, P3, D2	++
	23	P102L	MV	ND	M	61	50	+++	+++	T, D	T, D	S2, P3, D1	+++
	24	P102L	MM	2	F	65	64	+	++	T, D	T, D	S1, P3, D2	++
Figure 3E, F	25	P102L	MM	Negative	F	62	48	++	+	D	D	S2, P3, D1, PV1	+
	26	P102L	MM	Negative;8 kDa	M	52	155	+	+	D	D	P2	o
	27	P102L	MM	ND	F	66	81	+	+++	D	T, D	S1, P3	++
	28	6OPRI	MV	Negative	F	49	8	+++	+	T	D	S1	o
Figure 1E-H	29	6OPRI	MV	Negative	F	46	0	+++	+++	T	T, D	S2, P3, D3	o
	30	6OPRI	MM	ND	M	44	118	+++	+++	T, D	D	S3, GR1, P1, D1	+
Figure 3C, D, Figure 5A, B	31	6OPRI	MM	ND	F	45	72	+++	++	T	T, D	S1, PN1	+
	32	5OPRI	MM	ND	M	57	323	+	+	T, D	T, D	S1	+
	33	4OPRI	MM	ND	F	56	7	+++	+	T	T, D	S3, GR1	+
Figure 1A-D, Figure 3A, B	34	4OPRI	MM	ND	M	73	14	++	++	T, D	T, D	S1	+++
	35	4OPRI	MM	2	F	62	5	++	++	T	T, D	S1, GR1, PN1	++

*Proteinase K-digested PrP fragment size according to the London classification [14, 15].

ND, not done.

**Figure 1:**
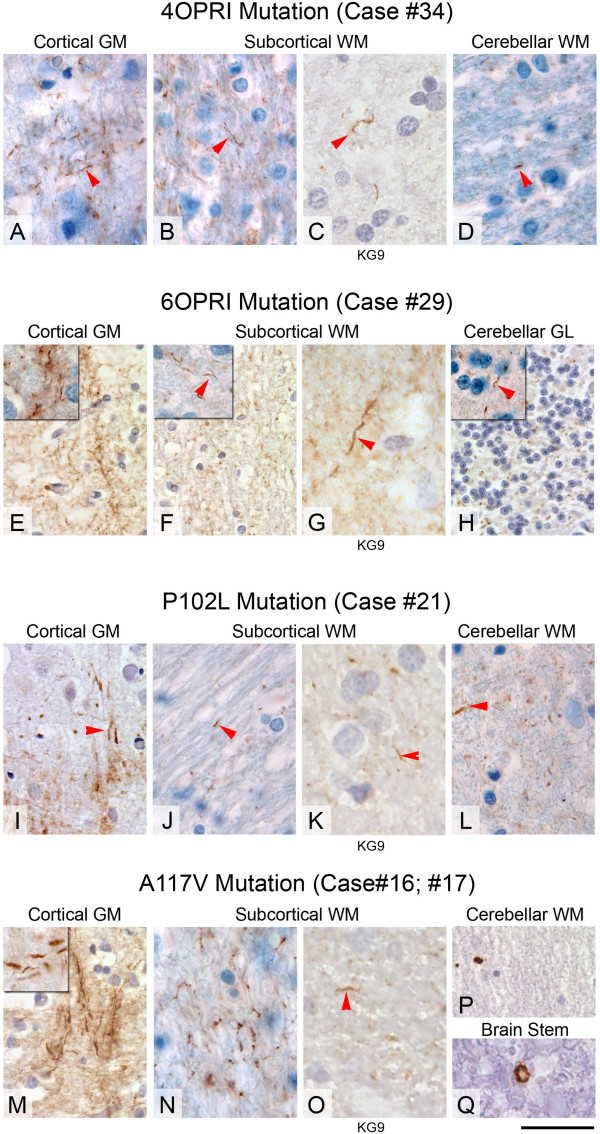
**Threads of PrP positive deposits in inherited prion diseases with 4 and 6 octapeptide repeat insert mutation, P102L (Gerstmann Sträussler-Scheinker syndrome), and A117V mutation. AD**, 4OPRI mutation with abundant threads, which form a dense crisscrossing network (corresponding to myelinated fibres) in the cortex (**A**) and forming multiple, thin parallel threads in the subcortical white matter, detectable with two antibodies, ICSM35 (**B**) and KG9 (**C**). Arrowheads in **B** and **C** point to accentuated threads. In the cerebellum (**D**), the PrP-containing filaments are thicker, shorter, and less abundant than in the cortex. The 6OPRI mutation (**E-H**) is characterised by abundant threads in the cortical grey matter I, subcortical white matter (**F**, ICSM35, **G**, KG9), and in the cerebellar granular layer (**H**). **I-L**, Both forebrain and cerebellum with *PRNP* P102L mutations show white matter threads of abnormal PrP in the subcortical (**J**, ICSM35 and **K**, KG9) or cerebellar (**L**) white matter. Arrows **I-L** show short threads of PrP deposition. Small myelinated fibres extending into the cerebral cortex also show threads of abnormal PrP (arrowhead in I) alongside with large amyloid plaques (not shown) and synaptic PrP. **M-Q**, A117V mutation (Case #16 M-P and Case #17, **Q**) with abundant cortical threads (**M**), corresponding to myelinated cortical fibres. The inset shows several parallel threads. Also the subcortical white matter is rich in PrP positive threads (**N**, ICSM35 and **O**, KG9). The cerebellum of the same case lacks filamentous inclusions, whilst small granular deposits are often observed (**P**). The brain stem of case #17 shows cross sections of myelinated fibres with occasional circular PrP-positive structures, corresponding to PrP containing myelin sheaths. Scale bar corresponds to 20 μm in P, 40 μm in the insets of **E**, **F**, **H** and **M**, to 80 μm in **C, G, H, O** and to 140 μm in all other panels.

**Figure 2:**
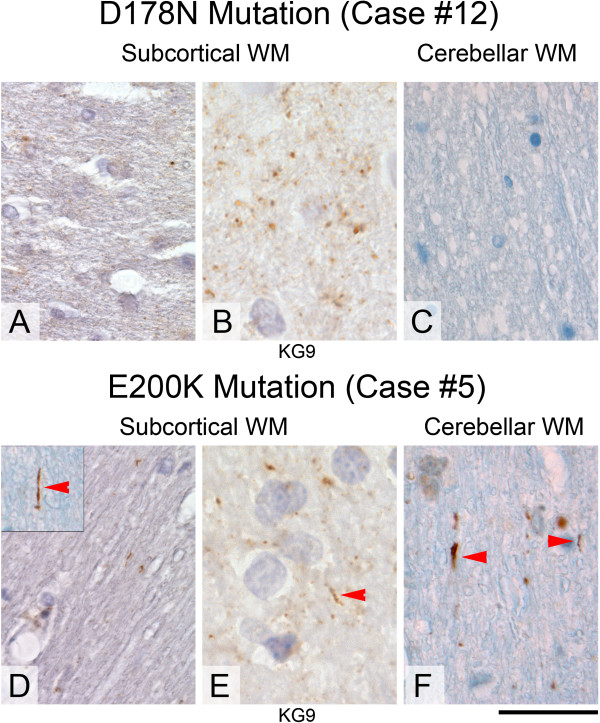
**Patterns of PrP deposits in brains with** ***PRNP*** **D178N and E200K mutations. A-C**, there are small dot like inclusions in the white matter of a case with D178N mutation (**A**, **B**, ICSM35 and KG9), no cerebellar threads (**C**). **D**-**F**, E200K mutation with sparse threads in the subcortical white matter (**D**, ICSM35 and **E**, KG9) and the cerebellar white matter (**F**). The inset in (**D**) shows a rare straight PrP positive filament and the arrowhead points at a short stub or dot of abnormal PrP. The cerebellar white matter (**F**) of the same case shows occasional thicker, straight filaments of abnormal PrP (arrowheads). Scale bar corresponds to 140 μm in (**D**), to 80 μm in **B, E** and to 40 μm in all other panels.

**Figure 3:**
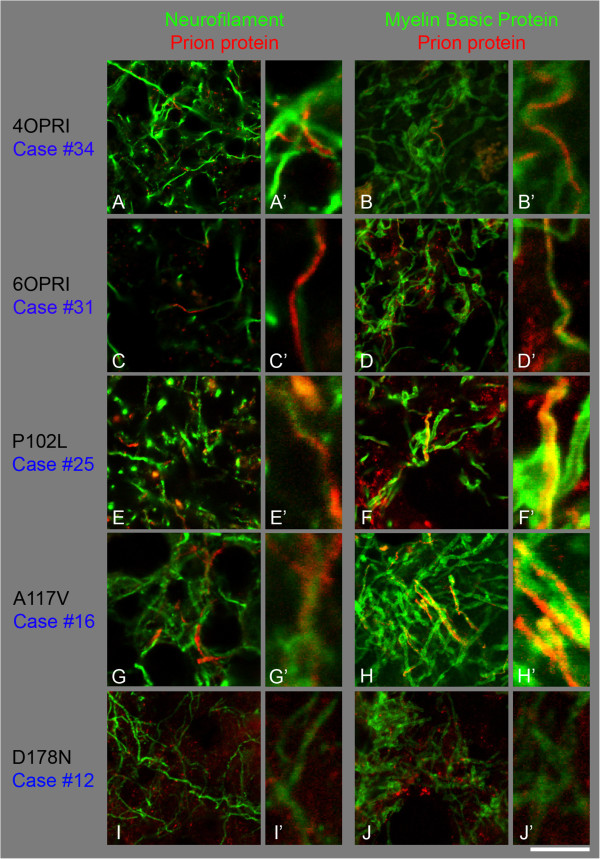
**Co-localisation studies of abnormal PrP (Alexa 546, red) with Neurofilament (A, C, E, G, I) or with myelin basic protein (B, D, F, H, J), (green, Alexa 488) in the white matter of the frontal lobe. A, B**, 4OPRI mutation shows the neurofilament signal being located beside abnormal PrP (**A, A’**), whereas there is an overlap between MBP and PrP signal (**B, B’**). **C, D**; 6OPRI with filamentous PrP being localised next to Neurofilament (**C, C’**) but directly congruent with MBP (**D, D’**). **E**, **F** there is little white matter PrP in this example of a P102L mutation, whilst a case with an A117V mutation (**G, H**) shows abundant white matter PrP, which shows PrP immunoreactivity next to neurofilaments, (**G, G’**) and co-localises with MBP, resulting in yellow signal (**H, H’**). The more C-terminal mutations, such as D178N show mostly granular PrP deposits and no strong filamentous PrP (**I, J**). Scale bar 16μm for **A-J** and 4 μm respectively for **A’**-**J’**.

**Figure 4:**
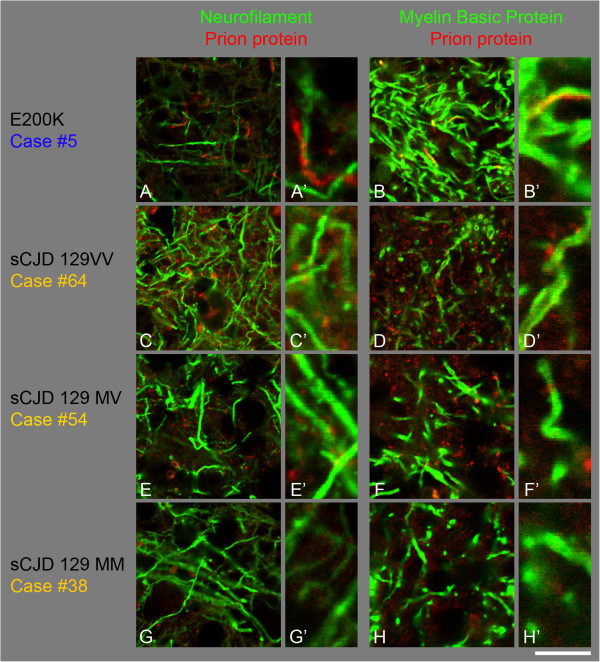
**Co-localisation studies of abnormal PrP (Alexa 546, red) with Neurofilament (A, C, E, G) or with myelin basic protein (B, D, F, H), (green, Alexa 488) in the C-terminal E200K mutation and in sCJD control cases (frontal cortex). A, B**, E200K mutations shows rare filamentous PrP, co-localising with the myelin sheath (**B**, **B’**) but not detectably with axonal neurofilament (**A, A’**). All sCJD cases (129VV, **C, D**; 129MV, **E**, **F**; 129MM, **G, H**) show variable amounts of granular PrP which does not co-localise with neurofilaments (**C, E, G**) or Myelin basic protein (**D, F, H**). Scale bar: 16 μm for **A-H** and 4 μm respectively for **A’-H’**.

**Figure 5:**
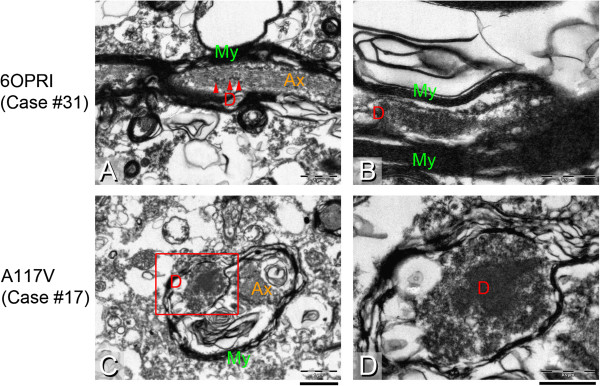
**Electron microscopy of two cases with abundant white matter deposition of PrP amyloid in the frontal cortex. A, B**, Case #31 with 6OPRI mutation shows small granular deposits in the axoplasm of a longitudinal section of a myelinated axon, and more substantial fibrillar electron dense deposits in a myelin sheath, that has become divided by these inclusions, raising the possibility of amyloid. **C**, **D**; Case #17 with an A117V mutation shows a cross section of a myelinated fibre with the axon (Ax) being separated from the para-axonal inclusion that led to a splitting of the myelin (My) sheath. The red box indicates the region shown in **D** with a characteristic electron dense deposition, raising the possibility of amyloid. Scale Bars: 1 μm (**A**, **C**), 0.5 μm (**B**, **D**).

**Figure 6:**
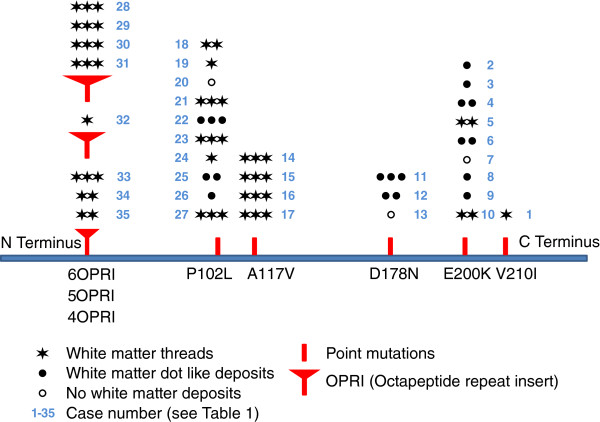
**Schematic representation of the deposition of white matter filamentous PrP in relation to the mutation: the horizontal bar represents the** ***PRNP*** **open reading frame with the N terminus on the left and the C terminus on the right.** OPRI and point mutations are indicated with red symbols. At each mutation, the cases with white matter threads (filaments) are indicated with stars; the number of stars indicates the abundance of deposits (corresponding to the score in Table 2). For cases where no filaments are seen, white matter dot-like deposits are indicated by filled circles and cases with no white matter deposits are symbolised by open circles. The blue number next to the symbols corresponds to the case numbers in Table 2, all figures and in the text). The position of the mutations on the schematic representation is not exactly to scale.

All case numbers (first column) are cross-referenced to Figures 1, 2, 3, 4, 5 and 6. The columns on the right describe the pattern of PrP deposition in the white matter. The semi quantitative density scores of white matter PrP are represented in column 8. mo: months, M: male/methionine, F: female, V: valine, WM: white matter, FB = Forebrain, Cer = Cerebellum, T = Threads, D = Dots. The two columns on the right describe the pattern and intensity of cortical PrP deposition as described in [11] and the degree of spongiform changes. S = synaptic, PN = perineuronal net, GR = Granular; P = well demarcated plaques, D = diffuse plaques; and PV = perivascular deposits. The number indicates the semi-quantitative intensity or density of the respective feature.

### Statistical analysis

The quantity score of thread pathology was transformed into numerical values (− = 0, + = 1, ++ =2, +++ =3. 4OPRI, 5OPRI and 6OPRI cases; and D178N, E200K, V210I cases, were grouped together to allow group sizes exceeding 3. The null hypothesis was the assumption of no difference between groups. Table 3 shows the results of the statistical analysis.

**Table 3:** **Calculation of the 1 way ANOVA**

Group	Number of subjects	Mean	Standard deviation
**1 (OPRI)**	8	2.5	0.88
**2 (P102L)**	10	1.7	1.53
**3 (A117V)**	4	3	0
**4 (E200K, D178N, V210I)**	13	1.4446	0.7697

## Results

35 cases of inherited prion disease were analysed, scored for PrP staining patterns and compared with 26 cases of sCJD. We observed changes in the grey and white matter that were similar to previously published patterns of abnormal PrP deposition. The specific findings and individual white matter staining pattern for each case with mutations in the *PRNP* open reading frame, relevant clinical data, codon 129 genotype, western blotting results, and cortical PrP pattern, are summarized in Table 2 and subsequently in this paragraph. In addition, a detailed description of the findings is given below. PrP^Sc^ molecular strain typing analysis was performed for about half the cases (Table 2). Where determined, molecular strain types were consistent with those published previously [15] however some brain samples were scored negative for PrP^Sc^ (Table 2). The apparent absence of detectable protease-resistant PrP is a relatively common feature of some forms of inherited prion disease and in part relates to sampling variation and in particular the region of brain studied [15]. Typically we examined only a single sample (~200 mg) of frontal cortex.

**Figure 7:**
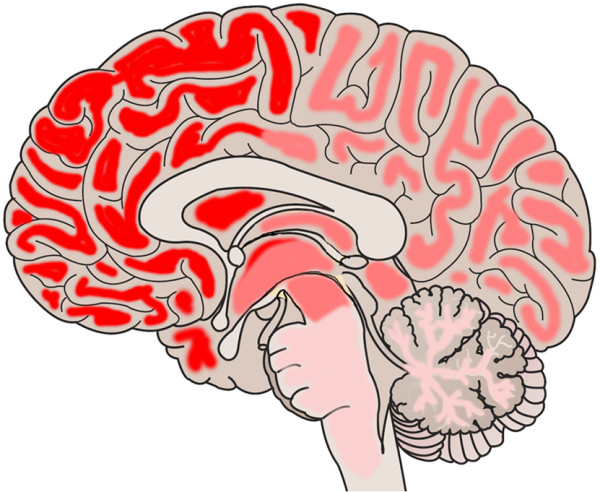
**Regional distribution of filamentous PrP in a brain with A117V mutation.** (Case #17): the intensity of the white matter filaments is represented in shades of red, the most intense representing a strong deposition (frontal, parietal and temporal brain) and there is a decreasing gradient towards the posterior lobes, the cerebellum and the brain stem, where fewer filaments were observed. The corresponding semi-quantitative evaluation is listed in Table 6.

The white matter of brains with inherited CJD prion disease show in the majority of cases filamentous PrP deposition in the frontal, temporal parietal and occipital regions of cerebral, subcortical white matter and to a much lesser extent in the cerebellar white matter, and the brain stem (Table 2, Figures 1, 2, 3, 4, 5, 6). Cortical areas that contain significant amounts of abnormal synaptic or plaque PrP, tend to show more abundant filamentous white matter whilst a low cortical PrP load is more often associated with very little or no filamentous PrP in subjacent white matter tracts. Table 4 and Figure 7 show the distribution of white matter filaments in Case #17. In this case, the strongest labelling is seen in frontal and temporal lobes, basal ganglia, followed by parietal and occipital regions, and the least frequent labelling in the cerebellum and pons. Occasionally we found circular PrP deposits in cross sections (Figure 1Q), suggestive of a localisation on myelin sheaths. In addition, there was fine granular or dot-like positivity in almost all cases, including sCJD of all three codon 129 genotypes (MM, MV and VV). When the cases with filamentous inclusions are plotted onto a diagrammatic representation of the *PRNP* open reading frame, we observed that the strongest and most consistent filamentous white matter deposits were in cases with N-terminal mutations (Figure 6), with octarepeat inserts, and A117V mutations, followed by P102L mutations. These filaments were much less frequent in brains with C terminal D178N, E200K and V210I mutations. The intensity was unrelated to disease duration or codon 129 genotype (Table 2) but appeared to mirror cortical PrP load. The two groups with marked formation of threads (4OPRI, 6OPRI and A117V) comprise younger patients (40-50ys); while patients with other mutations were more than 50 years old with the exception of two patients with an E200K mutation, of whom one showed an unusual clinical phenotype with very long disease duration (#5). The brain in this case showed filamentous white matter PrP. One way ANOVA confirmed that N terminal mutations are more likely to accumulate filamentous abnormal PrP than the white matter of C terminal mutant brains (Significance level p = 0.012). Instead, no filamentous, but variable granular PrP deposits are seen in cases of sCJD, with no intensity difference between the different polymorphisms on codon 129 (Table 5). Double labelling immunofluorescence studies confirm the presence of PrP filamentous material in the white matter. In keeping with light microscopy observations, the most abundant filaments are seen in 4- and 6OPRI, and A117V mutations, whilst P102L, D178N, E200K and V210L mutations are less often associated with filamentous PrP in the white matter. The filamentous nature and the localisation at light microscopy level are suggestive of axonal accumulation. To determine the precise location of the filaments in the white matter, we used double–labelling immunofluorescence and detection by confocal laser microscopy. There was virtually no overlap between Alexa 488 labelled neurofilaments and Alexa 546 labelled PrP, suggesting that the filamentous deposits are not in the axoplasm. Instead there were PrP threads that appeared to flank and run in parallel to axons, suggesting a localisation directly adjacent to the axoplasm of myelinated fibres. Indeed further co-localisation studies with Alexa 488 conjugated antibodies against myelin basic protein confirmed that these filamentous PrP deposits shows robust co-localisation of PrP threads with myelin sheaths of all sizes. Longitudinal sections of myelinated fibres occasionally contain PrP threads on either side of the centrally located axons (Figure 3). Sections of dual labelled axons show a co-localisation of PrP with MBP, sparing the central axon (Figure 3). In contrast, samples of sCJD contain granular deposits outside the myelin sheath but no filamentous PrP (Figure 4), in keeping with the observations by light microscopy.

**Table 4:** **Regional distribution of filamentous PrP in a brain with A117V mutation (Case #17): semiquantitative scoring of white matter PrP filaments**

Case #17: areas analysed	Density of filamentous PrP in white matter tracts in the region
Anterior frontal F1-F2	+++
Posterior frontal Superior frontal gyrus and pre motor cortex	++
Temporal T1-T2	+++
Temporal T2-T3	+++
Tip of temporal lobe	+++
Parietal	++
Occipital	++
Anterior cingulate	+++
Hippocampus	+++
Amygdala	+++
Basal ganglia, ant.	+++
Basal ganglia, mid.	+++
Basal ganglia, post	++
Thalamus with subthalamic nucleus	+
Upper midbrain	+/++
Lower midbrain	+
Pons	+
Lower medulla	n.a.
Cerebellum Vermis	+
Cerebellum with dentate	-
Cerebellum hemisphere	(+)

The graphical representation is shown in Figure 7.

**Table 5:** **Summary of intensity scores in sporadic CJD**

	Scores for granular PrP (number of cases)				
sCJD, codon 129 genotype	Score 0	Score 1	Score2	Score 3	Significance
129MM	1	3	3	2	MM vs. MV: 0.23 (n.s.)
129MV	3	4	2	1	MV vs. VV: 0.72 (n.s.)
129VV	2	2	2	1	MM vs. VV: 0.48 (n.s.)

The left columns indicated the genotype of the codon 129 polymorphism, the subsequent columns the number of cases with the indicated scores (0–3) and the right columns the statistical analysis of the scores between the different codon 129 genotypes. No statistically significant difference in the intensity scores are seen between cases with codon 129 MM, MV or VV.

In the following paragraphs we describe PrP deposits in cortical grey matter, followed by changes in the subcortical white matter, cerebellar grey and white matter changes and the co-localisation of abnormal PrP with myelin and axonal structures. Finally, the results of the dual labelling experiments are described. Type and intensity of the white matter deposits (none, dot like/granular or filamentous) with relation to the mutation are also summarised in a graphic representation in Figure 7. This figure also gives a reference to the cases in Table 2.

### 4OPRI [96 base pair insert] (1 male, 2 female); Figure 1A-D, Figure 3A, B

The mean age for this group is 63.7 years (56–73) (Cases #33-35). The cerebral grey matter of these cases shows both axonal threads and granular synaptic stain (Figure 1A). The white matter also shows threads and dots of abnormal PrP (Figure 1B,C). The cerebellar cortex displays the classical “striped” or “tigroid” pattern of PrP depositions, perpendicular to the cerebellar surface and the Purkinje cell layer as reported previously [11, 16] and the white matter comprises rare to moderately frequent dots and thread-like staining (Figure 1D). Filamentous deposits were seen with both antibodies (Figure 1B, ICSM35 and 1C, KG9). All three 4OPRI cases show consistent axonal labelling in cerebral grey and white matter as well as cerebellar white matter, in a pattern and intensity similar to that observed in the 6OPRI group [11, 17]. Co-localisation studies (Figure 3A,B) with PrP (red) and neurofilaments (green) show occasional parallel course of PrP threads and axons (Figure 3A,A’), while both longitudinal and transverse sections of myelin sheaths confirm the presence of the threads within the myelin structure Figure 3B,B).

### 5 OPRI [120 bp insert] (1 male)

The age of this patient was 57 years, with disease duration of several years (Case #32). The onset of his illness was difficult to determine as he gradually developed neurological symptoms in his forties which superimposed a pre-existing personality disorder with aggressiveness and outbursts. The brain showed PrP deposits that are similar to sporadic sCJD and the characteristic striping pattern, which is generally seen in the cerebellar cortex with 4OPRI and 6OPRI mutations, was absent. The cerebral white matter showed subtle white matter threads and there was no white matter PrP in the cerebellum.

### 6OPRI [144 bp insert] (1 male, 3 female); Figure 1E-H, Figure 3C,D, Figure 5A,B

The mean age for this group is 46 years (44–49) (cases #28-31). In all cases the cerebral grey matter contains abundant axonal threads (Figure 1E), as well as a granular synaptic stain and scattered microplaques. These threads are present in the myelinated fibres that radiate from the white matter into the cortex. The subcortical white matter contains PrP positive threads (Figure 1F, ICSM35 and 1G, KG9) and to a lesser extent, fine granules. The cerebellar molecular layer shows the classical, distinct “stripy” or “tigroid” pattern with an orientation perpendicularly to the cerebellar surface as described before [11, 18–20]. This pattern is a hallmark of octapeptide insert mutations and unlikely to be related to the filamentous deposits. Threads are also seen in the cerebellar granule layer (Figure 1H) and the cerebellar white matter shows variable amounts of dot- and thread-like staining. Case #30 shows a staining pattern that varies from the other cases with a 6OPRI mutation, in that the grey matter displays granular synaptic positivity without thread labelling and the white matter shows a predominance of granular labelling and fewer threads. In conclusion, this group shows consistent, strong filamentous positivity in both, grey and white matter (Figure 3), similar to the group with 4OPRI mutation (see above). The cerebellar white matter shows thread pathology, a feature also seen in cases with P102L and 4OPRI mutations. Co-localisation studies show a striking overlap of PrP with myelin sheaths, whilst colocalisation with axons was below detection limits, confirming that PrP threads are located extra-axonal, within the sheath (Figure 3C,D). However, ultrastructural studies of case #31 show granular protein deposits also in the axoplasm but, in keeping with immunofluorescence studies, more substantial deposits in the myelin sheath, which appears displaced and split by the amyloid aggregates (Figure 5A,B).

### P102L (6 male, 4 female); Figure 1I-L, Figure 3E, F

The mean age for this group is 60.8 years (53–66) (cases #18-27). The cerebral grey matter of these cases contains frequent core- and multicentric plaques with diffuse granular synaptic pattern as described before [21, 22] and occasional threads composed of abnormal PrP (Figure 1I). The subcortical white matter contains scattered plaques of small size with variable fine granular and thread-like positivity (Figure 1J, ICLM35 and 1K, KG9). The cerebellar cortex shows plaques and large granular aggregates. The white matter contains variable plaques, granules and short threads (Figure 1L). In the majority of cases with P102L mutation, a small, and at most moderate amount of filamentous labelling can be observed in the white matter and less dense axonal positivity in the grey matter. In more than two thirds of the cases filamentous labelling is also present in the cerebellar white matter. In immunofluorescence studies however, nearly no filamentous material was detected (Figure 3E,F).

### A117V (3 male, 1 female); Figure 1M-P, Figure 3G,H, Figure 5C,D

The mean age for this group is 43 years (40–47) (cases #14-17). The grey matter contains frequent granular plaques with diffuse, moderate-to-strong granular synaptic labelling as well as strongly labelled short (Figure 2M inset) and long filamentous white matter deposits (Figure 2M). In all three cases the white matter shows frequent and intense thread-like positivity with sparse fine granules and plaques (Figure 1N, ICLM35 and 2O, KG9). The cerebellar cortex mainly contains patches of plaques and the white matter shows occasional plaques with none or occasional dot-like PrP deposits as described before [23, 24]. In conclusion, this group displays a strong, dense filamentous PrP labelling in the cerebral white matter, similar to the 6OPRI and 4OPRI cases (see above), with significant filamentous PrP extending to the myelinated axons in the cortical grey matter and no cerebellar threads. A cross section of the pons (Figure 1Q) show occasional circular PrP deposits, strongly suggestive of a localisation in the myelin sheath. Immunofluorescent labelling confirms extensive co-localisation of PrP threads in the sheaths of myelinated fibres, and no axonal PrP. The dual labelling of PrP with neurofilaments demonstrates no colocalisation of the signal (Figure 3G, G’), whilst there is frequent and robust dual labelling of myelin sheaths and filamentous PrP (Figure 3H, H’). All A117V cases strongly accumulate filamentous PrP and it is generally very easy to detect filaments in the white matter of A117V cases. Ultrastructural studies of case #17 show substantial deposits of amyloid in the myelin sheath (Figure 4C,D).

### D178N (2 male, 1 female); Figure 2A-C, Figure 3I,J

The mean age for this group is 57 years (51–60); cases #11-13. Both patients showed clinical CJD phenotype. Here, the cortical grey matter shows diffuse, weak to moderate fine granular synaptic stain with plaques of variable size. The subcortical white matter shows a wide variability (none to very frequent) of fine granular positivity but no thread like labelling (Figure 2A, ICSM35 and 3B, KG9). The cerebellar cortex shows patchy synaptic stain and granular aggregates with variable amounts of condensed granular PrP, occasionally forming microplaques, and the subcortical white matter has very rare or PrP dot-like positivity (Figure 2C). Overall these cases show little PrP labelling in the white matter and no thread-like PrP deposits. No threads were detected by immunofluorescence, confirming the observations by light microscopy (Figure 3I,J).

### E200K (6 male, 3 female); Figure 2D-F, Figure 4A, B

The mean age for this group is 60 years (45–69) (cases #2-10). The cortical grey matter shows diffuse, granular or fine dot-like synaptic staining that ranges from weak to strong intensity, similar to that in sCJD. In addition, there was cortical perineuronal PrP deposition with variable amounts of microplaques, and there are rare filamentous PrP deposits. The subcortical white matter contains variable amounts (sparse to very frequent) of fine granular positivity (Figure 2D) and occasional white matter threads (Figure 2D, inset, ICSM35, and 2E, KG9). The cerebellar cortex shows patchy synaptic staining and granular aggregates with or without microplaques. The cerebellar white matter shows rare granular positivity and threads in some cases (Figure 2F). In one case (case 5 in Table 2) the cerebellar white matter showed frequent filamentous staining as well as dot-like positivity (Figure 2F, Figure 4A,B). This patient displayed an unusual clinical phenotype (younger age and significantly longer disease duration) compared to the other patients with the same mutation, which may explain the difference to the patients with older age and shorter disease duration. Overall, the PrP staining patterns in brains with the E200K mutation are similar to those observed in sCJD [25]. Among the cases with *PRNP* gene mutations, this is the only group that shows perineuronal PrP labelling. Double labelling immunofluorescence of one case (#5) shows filamentous PrP deposits. There is co-localisation of PrP in MBP positive myelin sheaths whilst PrP in axonal structures is below the detection limit (Figure 4A,B).

### V210I (1 male)

In this single case (#1) with this mutation, the findings are very similar to those in E200K mutations. We found only cerebral PrP^Sc^, some of which formed fine threads but no cerebellar PrP^Sc^. The cortex shows synaptic labelling with a homogenous distribution across all cortical layers, similar to that of sCJD.

### Sporadic CJD (26 cases), Figure 4C-H

26 cases with codon 129 polymorphism MM (9), MV (10) and VV (7) were examined. All cases showed variable amounts of granular PrP deposits, ranging from none to frequent, corresponding to a score from 0–3 (see Tables 5 & 6). Across all cases, no difference was seen in the intensities of granular white matter PrP deposition between codon 129 genotypes MM, MV and VV. Double labelling immunofluorescence of PrP with axons or myelin sheaths shows no co-localisation of granular white matter PrP with either structure in all three codon 129 genotypes. Instead there is frequent granular PrP, which is immediately adjacent to the myelinated fibres (Figure 4C-H).

**Table 6:** **List of sporadic CJD cases included in the control group**

Codon 129	Gender	Age (years)	Disease duration (Months)	Frequency of granular PrP deposits in the frontal subcortical white matter (intensity score)
MM	M	75	2.5	0
MM	M	76	3	2
MM	F	66	6	3
MM	M	89	13	1
MM	F	81	3	1
MM	M	62	3	2
MM	F	74	5	2
MM	F	63	3	3
MM	F	74	3	1
**Average MM**	**M:F = 4:5**	**73.3**	**4.6**	**1.67**
MV	F	73	7	1
MV	F	67	2	1
MV	F	70	10	0
MV	M	75	2	1
MV	M	62	11	0
MV	M	63	17	0
MV	M	56	11	2
MV	M	62	3	2
MV	M	77	12	3
MV	M	68	3	1
**Average MV**	**M:F = 7:3**	**67.3**	**7.8**	**1.1**
VV	M	69	9	0
VV	F	57	3	1
VV	F	72	5	1
VV	F	81	9	2
VV	F	81	3	0
VV	M	83	3	2
VV	M	61	2	3
**Average VV**	**M:F = 3:4**	**72**	**4.8**	**1.29**

Cases with Codon 129MM, MV and VV alleles were used. The table also lists the age at death, duration of illness and the density of granular PrP in the white matter. The case numbers are cross-referenced in Figure 4.

## Discussion

Our study shows that filamentous white matter PrP deposition correlates with the localisation of the *PRNP* mutation and to some extent with the age of the patients. Previous studies have described PrP plaques in the CNS white matter in various forms of prion diseases including vCJD [6] and some cases of 129MM sCJD [7] and in the cerebellum of 129VV patients [26]. A study of 20 autopsy cases of CJD (not further specified), revealed PrP^Sc^ deposition in both cerebral and cerebellar white matter in 4 cases [8]. Immunohistochemical detection of abnormal PrP showed structures described as “arrays adjacent to ‘myelinic’ fibres and as clumps adjacent to oligodendrocyte nuclei”, and transmission electron microscopy showed that they were associated with “dense lysosomes in oligodendroglial perikarya and in their processes”. These inclusions were characterized as “finely fibrillary, paracrystalline, amorphous, or densely osmophilic material”. The authors concluded that white matter involvement in spongiform encephalopathy may be due to direct modifications of oligodendroglial cells associated with abnormal metabolism of PrP [8]. In inherited prion diseases, white matter plaques have been described before, and typically present as small, well circumscribed amyloid deposits [21, 24].

In animals, PrP accumulation in the white matter has been described in sheep with atypical scrapie, with two staining patterns; a ‘globular’ type, forming ring- or oval-shaped deposits prominent in the spinocerebellar tracts, reticular formation, cerebellar and cerebral white matter, and other white matter tract areas and a ‘punctate’ type consisting of small circle or elongated tear-drop deposits prominent in several grey matter areas, e.g. the reticular formation, substantia nigra, fimbria hippocampi, and amygdala [9]. Again these features are distinct from the filamentous immunoreactivity in human inherited prion disease. Experimental models of prion disease have added only little insight into the possible pathogenesis of white matter PrP deposition. In a study of scrapie infected Syrian hamsters [10], an intense PrP^Sc^ signal was found in the white matter, and was thought to support the hypothesis that PrP^Sc^ is transported along axons, but the histoblot technique had a resolution insufficient for the subcellular localisation of the deposits. Studies using the mouse adapted Fujisaki prion strain reported white matter destruction and deposition of abnormal PrP in white matter tracts [8, 27], without being more specific regarding the PrP deposition pattern. The CJD Fujisaki strain was derived from a brain that now is regarded as a case of P102L GSS [27].

Several transgenic mouse models of inherited prion disease have been generated but no specific white matter pathology has been reported. A transgenic mouse model expressing a nine-octapeptide insertion mutant mouse PrP (9OPRI) fused with enhanced green fluorescent protein [28] exhibited a striking white matter accumulation of abnormal PrP with intra-axonal mutant PrP as linear filamentous aggregates in central and peripheral axons, suggesting disruption of axonal transport [28]. Several models of the human P102L feature plaques but no white matter PrP [29–34]. Transgenic mice expressing a murine *Prnp* A116V (corresponding to the human *PRNP* A117V) mutation [35] on a *Prnp*^*0/0*^ background displays predominantly plaque pathology, chiefly in the cerebellum. Mice expressing the homologue of the human D178N (129V) mutation showed motor dysfunction, alteration of spatial working memory and abnormal EEG pattern and sleep disturbances [36], histologically punctate, micro-plaque like deposits, and ultrastructurally a swelling of the endoplasmic reticulum of cerebellar granule neurons. A model of the E200K mutation [34, 37] showed no spontaneous disease even after 900 days. Inoculation with human E200K -129VV brain yielded extensive plaques while an E200K 129MM inoculum yielded a predominantly synaptic pattern, but no white matter pathology was observed [34].

Previous studies of the transport of PrP along axonal pathways in animal models have concluded that transport of infectious prions uses mechanisms distinct from fast axonal transport [38], in contrast to PrP^C^[39]. It has been suggested that prion spread occurs via non-canonical mechanisms [40, 41], for example in a domino-like manner along PrP^C^ expressing nerve membranes or Schwann cells [42]. It is possible that an impairment of axonal transport or clearing mechanisms leads to deposition in the myelin sheath of N-terminal mutant PrP. Whilst no quantitative (and comparative) data exist for the amount of PrP^C^ in CNS oligodendrocytes and axons, experimental data of the peripheral nervous system suggest that Schwann cells express negligible levels of *Prnp* mRNA, and that expression of axonal PrP is required for Schwann cell myelin maintenance [43]. It is possible but remains speculative that a similar mechanism, involving transfer of PrP from the axon to the myelin sheath is relevant for the maintenance of CNS myelin. It is possible that soluble forms or micro-aggregates of PrP, produced in the neuronal body, move by passive or active axonal transport. Our ultrastructural studies provide evidence of granular protein deposits in the axoplasm, possibly representing abnormal PrP, which may eventually accumulate in myelin sheaths. Indirect evidence for this suggestions comes from the observation that the most abundant filamentous white matter PrP is localised in the vicinity of cortical areas that contain significant amounts of abnormal synaptic or plaque PrP, whilst a low cortical PrP load is more often associated with very little or no filamentous PrP in subjacent white matter tracts.

Filamentous PrP deposits are not observed in sCJD. A possible explanation is the different pathogenesis, in that all neuronal cells express mutant PrP and that all cells carrying the mutation may synchronously convert mutant PrP into the abnormally folded form of PrP. The variability between cases with the same mutation (see [22]) may be explained by a variable capacity to clear abnormal PrP, or a differential propagation of disease-related isoforms, as for example shown for the P102L mutation, where at the molecular level three isoforms of protease-resistant PrP with divergent physicochemical properties can be propagated [22]. The difference between cases with different mutations can be explained by different propensities to form deposits, as well as a region-specific propensity of cell populations to accumulate abnormal PrP even within the same family. It could also be argued that the initiation of the disease as well as the mechanism of cell-to-cell transmission in sporadic prion diseases is different from inherited forms. A remarkable observation is a very strong bivariate correlation between age of the patient at death and the density of threads in the white matter (p = 0.001). A regression model even favours age over mutation type, that is, age is a stronger determinant of the density of threads than the mutation itself (patients with more threads die younger). However, this correlation is explained by the fact that age at death correlates with the mutation.

Functional studies are required to further elucidate the mechanism of PrP deposition in the myelin sheath, and the propensity of N-terminal mutations to form such inclusions.

## Conclusion

We report here the presence of white matter deposition of abnormal PrP in inherited prion diseases. Strikingly, abnormal PrP appears to deposit in the myelin sheath of myelinated axons. The filamentous inclusions are strongest in cases with N-terminal mutations (OPRI, A117V) and less intense or absent in mutations toward the C-terminus (D178N, E200K). Areas that are most affected are in the frontal, temporal and parietal lobes and less filaments are seen in the occipital lobes, the cerebellum and the brain stem.
